# Editorial: Critical Care After Stroke

**DOI:** 10.3389/fneur.2022.903417

**Published:** 2022-04-13

**Authors:** Rajiv Advani, Roland Faigle, Sang-Bae Ko

**Affiliations:** ^1^Stroke Unit, Department of Neurology, Oslo University Hospital, Oslo, Norway; ^2^Neuroscience Research Group, Stavanger University Hospital, Stavanger, Norway; ^3^Department of Neurology, Johns Hopkins University School of Medicine, Baltimore, MD, United States; ^4^Department of Neurology, Department of Critical Care Medicine, Seoul National University College of Medicine, Seoul, South Korea

**Keywords:** stroke, intracerebral hemorrhage, ischemic stroke, acute medicine, critical care, thrombolysis (tPA), thrombectomy, endovascular therapy (EVT)

Stroke, as an entity comprising both acute ischemic stroke (AIS) and Intracerebral Hemorrhage (ICH), is the second leading cause of global mortality (1). Although advances in the management of AIS, in particular endovascular therapy for large vessel occlusion, have led to improved functional outcomes (2), 3-month mortality in those undergoing treatment remains substantial. Despite surgical intervention and changes in recommendations for acute medical management for ICH in recent years (3), 30 day mortality is as high as 46% (4). *Critical Care After Stroke* is therefore of upmost importance to improve functional outcomes and reduce mortality in both the short term and longer term follow up.

Endovascular Therapy (EVT) is now the standard of care for patients presenting with a large vessel occlusion in the anterior circulation (5), blood pressure management thereafter, however, is not standardized. In their mini review, Peng et al. address one of the elephants in the room, namely that, despite successful reperfusion, many patients do not regain functional independence. Their review identifies blood pressure (BP) optimization as an area of focus in those with hemodynamic variability and vast BP fluctuations. Ongoing trials are addressing the role of BP, but *post-hoc* analyses from several thrombectomy trials suggest that high systolic BP trajectories in the first 24 h post procedure are associated with an increased risk of poor outcome. An individual, autoregulation-guided approach to BP, seems to increase the chances of a good clinical outcome.

Hong et al.'s review on hemorrhagic transformation after an AIS highlights the associated risk of poor outcome and increased mortality. The mechanism of hemorrhagic transformation is explained by disruption of the blood-brain barrier and reperfusion injury that leads to leakage of peripheral blood cells. In AIS this transformation may be a natural progression of tissue ischemia that is facilitated, and thus worsened, by reperfusion therapy. There are several strategies that can be considered for management of hemorrhagic transformation in AIS, including neurosurgical intervention and medical management. The medical management is individual and can be summarized as reversal of coagulopathy, management of BP, temperature regulation, and supportive neurocritical care with a focus on reducing hematoma expansion and maintaining the integrity of the blood-brain barrier. Regarding the latter, the authors point out the role of matrix metalloproteinases and the need for more research around these biological and molecular mechanisms.

Kobata et al. review recent updates in neurosurgical interventions for spontaneous ICH. Neurosurgical interventions are a matter of debate and clinical practice varies greatly globally. The minimally invasive surgery plus alteplase for intracerebral hemorrhage evacuation (MISTIE) III trial demonstrated a reduction in mortality compared to medical treatment. Despite the overall reduction in mortality, no improved functional outcome categorized as a modified Rankin Scale 0–3 was observed. Ongoing randomized controlled trials such as the Early miNimally invasive Removal of Intra-Cerebral Hemorrhage (ENRICH) trial, the Minimally Invasive Endoscopic Surgical Treatment with Apollo/Artemis in Patients with Brain Hemorrhage (INVEST) trial, and the Dutch Intracerebral Hemorrhage Surgery Trial (DIST) are further addressing the optimal treatment for ICH. The authors point out that the outcome of surgical treatment is also dependent on the site and surgeon experience, something that should be taken into consideration when interpreting results for clinical practice.

Gao L. et al. address end-of-life care and the underlying decision-making process and associated prognostic uncertainties as a pivotal part of a stroke physician's challenge in the clinical setting. Patients suffering a stroke, especially those who require critical care, are commonly unable to actively participate in the care decision-making processes, and care decisions rest on the shoulders of surrogate decision-makers. Decision-making around many aspects of critical care can be challenging; surgical interventions, intensive care unit treatment, artificial nutrition, tracheostomy, withdrawal of life-sustaining care to name a few. This mini review highlights the difficulties in outcome prognostication, and provide strategies to address uncertainty and elicit goals of care. The authors conclude that clear communication regarding decision-making, prognostication, and patients' and surrogates' wishes after stroke are pivotal in all care settings, but especially in the critical care unit.

This topic also includes publications on the evaluation of the patient safety climate in acute care (Bohmann et al.), the impact of blood pressure and volume contraction in acute stroke (Bahouth et al.), early initiation of renal replacement therapy in ICH (Schenk et al.), reduction of intracranial pressure mediated through surgical intervention (Al-Kawaz et al.), individual predictors of mortality in ICH (Gao B. et al.; Sun et al.), and Subarachnoidal hemorrhage (Yang et al.; He et al.), the impact of atrial fibrillation Wu et al. and prolonged QT interval Ahn et al. on clinical outcomes in AIS patients, as well as other reports of original research showcasing the diversity in critical care by Mazza et al. and Nguyen et al.

The publications in this fascinating Research Topic have highlighted its multifaceted nature and comprise molecular and biological mechanisms, epidemiology, reviews of current literature of hot topics in the field of stroke care, outcome prediction, and prognostication and communication to name a few. The publications also point out areas for further research. This Research Topic highlights that *Critical Care After Stroke* poses substantial clinical challenges in a rapidly evolving area of stroke research.

## Author Contributions

RA drafted the manuscript. RF and S-BK contributed with conceptualization and editing of the final version. The final version was approved by all authors.

## Conflict of Interest

The authors declare that the research was conducted in the absence of any commercial or financial relationships that could be construed as a potential conflict of interest.

## Publisher's Note

All claims expressed in this article are solely those of the authors and do not necessarily represent those of their affiliated organizations, or those of the publisher, the editors and the reviewers. Any product that may be evaluated in this article, or claim that may be made by its manufacturer, is not guaranteed or endorsed by the publisher.

## References

[1] Collaborators GUND Feigin VL Vos T Alahdab F Amit AML Barnighausen TW. Burden of neurological disorders across the US from 1990-2017: a global burden of disease study. JAMA Neurol. (2021) 78:165–76. 33136137 10.1001/jamaneurol.2020.4152 PMC7607495

[2] Goyal M Menon BK van Zwam WH Dippel DW Mitchell PJ Demchuk AM. Endovascular thrombectomy after large-vessel ischaemic stroke: a meta-analysis of individual patient data from five randomised trials. Lancet. (2016) 387:1723–31. 10.1016/S0140-6736(16)00163-X 26898852

[3] Advani R Sandset EC. Insights into a personalized management of blood pressure in acute stroke. Curr Opin Neurol. (2022) 35:39–44. 10.1097/WCO.0000000000001016 34845148

[4] Zahuranec DB Lisabeth LD Sanchez BN Smith MA Brown DL Garcia NM. Intracerebral hemorrhage mortality is not changing despite declining incidence. Neurology. (2014) 82:2180–6. 10.1212/WNL.0000000000000519 24838789 PMC4113463

[5] Casaubon LK Boulanger JM Blacquiere D Boucher S Brown K Goddard T. Canadian stroke best practice recommendations: hyperacute stroke care guidelines, update 2015. Int J Stroke. (2015) 10:924–40. 10.1111/ijs.12551 26148019

